# Prognosis of patients with dementia: results from a prospective nationwide registry linkage study in the Netherlands

**DOI:** 10.1136/bmjopen-2015-008897

**Published:** 2015-10-28

**Authors:** Irene E van de Vorst, Ilonca Vaartjes, Mirjam I Geerlings, Michael L Bots, Huiberdina L Koek

**Affiliations:** 1 Julius Center for Health Sciences and Primary Care, University Medical Center Utrecht, Utrecht, the Netherlands; 2 Department of Geriatrics, University Medical Center Utrecht, Utrecht, the Netherlands

**Keywords:** GERIATRIC MEDICINE

## Abstract

**Objective:**

To report mortality risks of dementia based on national hospital registry data, and to put these risks into perspective by comparing them with those in the general population and following cardiovascular diseases.

**Design:**

Prospective cohort study from 1 January 2000 through 31 December 2010.

**Setting:**

Hospital-based cohort.

**Participants:**

A nationwide hospital-based cohort of 59 201 patients with clinical diagnosis of dementia (admitted to a hospital or visiting a day clinic) was constructed (38.7% men, 81.4 years (SD 7.0)).

**Main outcomes and measures:**

1-year and 5-year age-specific and sex-specific mortality risks were reported for patients with dementia visiting a day clinic compared with the general population; for patients hospitalised with dementia compared with patients hospitalised for acute myocardial infarction (AMI), heart failure or stroke, these were presented as absolute and relative risks (RRs).

**Results:**

1-year mortality was 38.3% in men and 30.5% in women. 5-year risk was 65.4% and 58.5%, respectively. Mortality risks were significantly higher in patients with dementia admitted to the hospital than in those visiting a day clinic (1-year RR 3.29, 95% CI 3.16 to 3.42; and 5-year RR 1.79, 95% CI 1.76 to 1.83). Compared with the general population, mortality risks were significantly higher among patients visiting a day clinic (1-year RR for women 2.99, 95% CI 2.84 to 3.14; and for men 3.94, 95% CI 3.74 to 4.16). 5-year RRs were somewhat lower, but still significant. Results were more pronounced at younger ages. Mortality risks among admitted patients were comparable or even exceeded those of cardiovascular diseases (1-year RR for women with dementia vs AMI 1.24, 95% CI 1.19 to 1.29; vs heart failure 1.05, 95% CI 1.02 to 1.08; vs stroke 1.07, 95% CI 1.04 to 1.10). 5-year RRs were comparable. For men, RRs were slightly higher.

**Conclusions:**

Dementia has a poor prognosis as compared with other diseases and the general population. The risks among admitted patients even exceeded those following cardiovascular diseases.

**Strengths and limitations of this study**

- The very large sample size and complete follow-up of all included patients.
- We had the unique opportunity to put the mortality risks of patients with dementia into perspective by comparing these with other diseases affecting the elderly.
- Except for absolute mortality risks according to age, sex, setting and type of dementia, other patient characteristics were not taken into account (eg, level of education, severity of dementia and comorbidity). Therefore, we cannot conclude that the differences in age-specific and sex-specific prognosis between dementia and cardiovascular disease can be entirely attributed to the dementia condition. However, such a causality reasoning was beyond the scope of this particular study.

## Introduction

Dementia is a severe disease with often a poor prognosis. Mortality risks are estimated to be at least two times higher than mortality risks in non-demented patients.^1^ Furthermore, it is expected that dementia will be among the leading causes of death in the near future instead of cardiovascular diseases (CVDs).^2^ ^3^ Survival time, however, ranges considerably between patients^4–7^ and ultimately depends on underlying risk factors, including age, sex and comorbid conditions. Studies focusing on the relation between these different factors and prognosis following a diagnosis of dementia showed inconsistent results.^8^ ^9^ While a number of studies have found worse survival time in men^4^ ^6^ ^10^ ^11^ and at higher ages,^6^ ^7^ others have found no association or even a reverse relation.^7^ ^12^ In addition, whether the type of dementia affects prognosis is not clear. Some studies found a shortened survival time in patients diagnosed with vascular dementia (VaD) compared with Alzheimer's disease (AD),^9^ while others found no differences.^13^ ^14^ These inconclusive results might be partly explained by the fact that most of these studies were small, used a selected group of patients (eg, nursing home residents with advanced dementia), had different follow-up times ranging from 0.2 to 15 years, and estimated survival time either from onset of symptoms or from time of diagnosis which makes generalisability limited. One large, methodologically sound study by Garcia-Ptacek *et al*^15^ showed that male gender and age were associated with increased mortality, with lowest risks seen in patients with AD.

Information on life expectancy can be valuable for patients, caregivers and clinicians in decision-making concerning diagnostic interventions, therapy and advanced care planning.^16^ Decision-making in clinical practice is inevitably dependent on expected prognosis. Information on prognosis also is crucial in developing and maintaining preventive strategies. Since robust data on absolute age-specific and sex-specific mortality risks in large cohorts are limited, estimations on prognosis of dementia from large population studies are needed.

The aim of this study was to report age-specific and sex-specific mortality rates of patients with dementia and its two most common subtypes, AD and VaD, in a large nationwide Dutch hospital-based cohort. To put these mortality rates into perspective, we also compared those at the day clinic with the general population and with those admitted with dementia with other diseases among specific subpopulations. Since CVDs are a leading cause of death in Western countries, we compared mortality risks of patients with dementia with mortality risks among patients hospitalised for stroke, acute myocardial infarction (AMI) and heart failure.^17^

## Methods

### Databases

To construct a cohort of patients with dementia, information from three databases was linked, the Dutch Hospital Discharge Register (HDR), the Dutch Population Register (PR) and the National Cause of Death Register. Since the 1960s, medical and administrative data for all admitted and day clinic patients visiting a Dutch hospital are recorded in the HDR; no information from outpatient visits and nursing home residents is available. Patients in the Netherlands are referred to the day clinic either in case of memory-related disorders or with multimorbidity. In the Netherlands, a day clinic visit is a 1-day hospital admission and therefore, considered to be inpatient care. Around 100 hospitals participate in the register. The HDR contains information on patients’ demographics (date of birth, gender), type of hospital, admission data, and principal and secondary diagnoses at admission. The principal and secondary diagnoses are determined at discharge and coded using the ninth revision of the International Classification of Diseases (ICD-9-CM).^18^ The PR contains information on all legally residing citizens in the Netherlands, including date of birth, gender, current address, postal code, nationality and native country. In the National Cause of Death Register, all primary and any underlying causes of death are reported. In the Netherlands, it is mandatory to complete a death declaration form after the death of any person, which has to be reported in the national cause of death statistics. Death reports are coded according to the International Statistical Classification of Diseases and Related Health Problems, 10th version.^19^ The overall validity of these registries has been shown to be high.^20^

### Cohort identification

To construct a cohort of patients with dementia first ever hospitalised or first ever referred to the day clinic for dementia, all patients with either a principal or secondary diagnosis of dementia (ICD-codes 290.0; 290.1; 290.3; 290.4; 294.1; 331.0; 331.1; 331.82) aged between 60 and 100 years were selected from the HDR between 1 January 2000 and 31 December 2010. Patients with a previous admission with principal or secondary diagnosis of dementia during the period 1 January 1995 until 1 January 2000 were excluded.

In the Dutch population, there are about 2.9 million people age 60 years and older. A recent validation study performed in our hospital showed high validity of the use of ICD-9 codes to identify patients with dementia (positive predictive value was 93.2%), and the two most common subtypes AD and VaD (positive predictive value was 63.2% and 91.3%, respectively).^21^ Following individuals over time based on information from the HDR is difficult as different hospital admissions of the same person cannot be recognised adequately, for example, if a patient was admitted in another hospital. Therefore, the collected cases were linked with the PR by using the record identification number assigned to each resident in the Netherlands with a unique combination of date of birth, sex and the numeric part of the postal code. The use of the unique record identification number enables to identify different admissions, even in different hospitals, by the same person. Through linkage of these selected cases with the National Cause of Death Registry, follow-up information on date of death, and the principal and underlying causes of death could be obtained. Information on severity of disease, presence of risk factors or medication use was not available in the registry. The approach resulted in a cohort consisting of 59 201 patients.

### Privacy issues

Linkage of data from the different registries was performed in agreement with the privacy legislation in the Netherlands.^22^ Only anonymised records and data sets are involved. The study did not have to be assessed according to the regulations of the research complying with the Dutch law on Medical Research in Humans. All linkages and analysis were performed in a secure environment of Statistics Netherlands.

### Data analysis

Continuous data were summarised as mean and SD, or as median and IQR where appropriate. Categorical data were summarised as percentages. Patients with dementia were followed up from their earliest date of hospitalisation or day clinic visit. Follow-up time ended on 31 December 2010 or earlier if a patient had died before end of the study. Mortality follow-up was complete for the entire cohort up to 31 December 2010.

First, absolute mortality risks in patients with dementia in two different time periods were examined: from admission/day clinic visit to 1 year thereafter, and from admission/day clinic visit to 5 years thereafter. Second, relative risks (RRs) were calculated to compare mortality risks for men versus women, presented with corresponding 95% CIs. Similar analyses were performed for the two most common dementia subtypes (AD and VaD). Third, we calculated RRs with corresponding 95% CI for patients admitted to the hospital versus patients visiting the day clinic. Fourth, since we expected differences in prognosis between patients visiting a day clinic and those hospitalised with dementia, we divided the cohort into two groups (patients visiting a day clinic and hospitalised patients). Mortality risks of patients with dementia visiting a day clinic were compared with mortality risks of the general population. Age-specific and sex-specific 1-year mortality risks for the general population of men and women aged 60–99 years, and 5-year mortality risks of individuals aged 60–94 years were available online from Statistics Netherlands.^23^ A direct method for age standardisation was used on the basis of the age distribution of the 2005 Dutch population with 5-year age groups. RRs were calculated (dementia vs general population) with 95% CI.

Finally, we compared mortality risks of patients hospitalised for dementia with mortality risks in other disease-specific subpopulations. These disease-specific subpopulations comprised patients admitted to a hospital with CVDs, affecting older patients in particular (AMI, heart failure and stroke). Absolute risks of CVDs were obtained from previous nationwide register linkage cohort studies, all using data from the HDR.^24^ ^25^

Data were analysed with SPSS software, V.20.0 (SPSS Inc, Chicago, Illinois, USA). A two-sided p value <0.05 was considered statistically significant.

## Results

In total, 59 201 patients (38.7% men) were identified through record linkage of the HDR with the PR and the National Cause of Death Register. Mean age was 81.4 years (SD 7.0). Number of patients per year of admission ranged from 4144 in 2000 to 8204 in 2010. A majority (62.4%) was diagnosed with AD, 12.5% with VaD. One-third of the cohort (37.0%) had a principal diagnosis of dementia. In those with a secondary diagnosis, principal admission reasons were, for example, bone fractures (12.0%), CVDs (8.1%) and pneumonia (8.0%). Baseline characteristics are shown in table 1.

**Table 1:** Characteristics of patients with a first hospitalisation or day/memory clinic visit for dementia in the Netherlands between 2000 and 2010

	Men	Women	Total
Number of patients	22 936	36 265	59 201
Age (years)			
Mean (SD)	79.9 (7.0)	82.4 (6.8)	81.4 (7.0)
Type of admission (%)			
Day clinic	31.2	31.8	31.6
Inpatient care	68.8	68.2	68.4
Origin (%)			
Native	91.7	90.9	91.2
Follow-up			
Median days (95% CI)	594 (576.4 to 611.6)	882 (864.7 to 899.3)	761 (748.3 to 773.7)
Dementia diagnosis			
AD	58.4	65.0	62.4
VaD	15.8	10.5	12.5
Other	25.8	24.5	25.1

AD, Alzheimer's disease; native, both parents born in the Netherlands; VaD, vascular dementia.

### One-year mortality risk among patients with dementia

One in every three women (30.5%) and men (38.3%) died within 1 year following a first hospitalisation or day clinic visit for dementia (table 2). Mortality risks increased in older age groups and were significantly higher in men than in women (RR 1.26, 95% CI 1.23 to 1.28) across all age groups. Among women who died within 1 year, the median survival time was 75 days, IQR 26–183.5, and for men 68 days, IQR 23.5–175.0.

**Table 2:** One-year and 5-year mortality risk in patients with a first hospitalisation or day/memory clinic visit for dementia in the Netherlands between 2000 and 2010, by age and sex

Age, years	Women, n	Men, n	Women, % deaths	Men, % deaths	RR (95% CI) for men vs women
1-year mortality					
60–64	537	650	11.7	17.5	1.49 (1.12 to 1.99)
65–69	1032	1282	14.1	22.1	1.57 (1.31 to 1.89)
70–74	3034	2909	19.2	26.6	1.38 (1.26 to 1.52)
75–79	6651	5460	21.8	33.3	1.53 (1.44 to 1.62)
80–84	10 317	6510	28.7	41.5	1.45 (1.39 to 1.51)
85–89	9639	4525	35.9	47.1	1.31 (1.26 to 1.37)
90–94	4203	1391	46.2	59.0	1.28 (1.21 to 1.35)
95–99	852	209	54.1	65.6	1.21 (1.08 to 1.36)
Total	36 265	22 936	30.5	38.3	1.26 (1.23 to 1.28)
5-year mortality					
60–64	537	650	25.5	34.3	1.34 (1.12 to 1.61)
65–69	1032	1282	34.2	45.6	1.33 (1.20 to 1.48)
70–74	3034	2909	44.2	53.6	1.21 (1.15 to 1.28)
75–79	6651	5460	48.7	62.6	1.29 (1.24 to 1.33)
80–84	10 317	6510	57.2	70.0	1.22 (1.20 to 1.25)
85–89	9639	4525	65.9	74.4	1.13 (1.11 to 1.15)
90–94	4203	1391	76.2	81.3	1.07 (1.03 to 1.10)
95–99	852	209	79.6	82.3	1.03 (0.96 to 1.11)
Total	36 265	22 936	58.5	65.4	1.12 (1.10 to 1.13)

RR, relative risk.

Similar findings were found for dementia subtypes (AD and VaD). In total, 7968 of 23 566 women with a first hospitalisation or day clinic visit for AD (33.8%) died within 1 year after diagnosis. In men, this percentage was significantly higher; 5571 of 13 389 (41.6%) died (RR for men vs women: 1.23, 95% CI 1.20 to 1.26). In patients diagnosed with VaD, 39.5% of men and 29.2% of women died (RR for men vs women: 1.35, 95% CI 1.27 to 1.44). Absolute mortality risks and RRs stratified by age and sex are presented in online supplementary appendix A for AD and in online supplementary appendix B for VaD. The overall age-adjusted RR for VaD versus AD was 0.98, 95% CI 0.96 to 1.00 (data not shown).

Table 3 shows absolute and RRs in patients following a first hospitalisation versus patients with a first day clinic visit for dementia. Forty-three per cent of patients admitted to the hospital died within 1 year (median survival time was 63 days, IQR 22–161), whereas 13.1% of patients visiting a day clinic died (median survival time was 165 days, IQR 78–262). Overall, short-term mortality risks were higher in patients admitted to the hospital (RR for inpatients vs patients visiting a day clinic: 3.29, 95% CI 3.16 to 3.42), particularly in the youngest patients (highest RR in patients aged 60–64 years: 7.40, 95% CI 4.61 to 11.88).

**Table 3:** One-year and 5-year relative mortality risk in patients with a first hospitalisation versus a first day/memory clinic visit for dementia in the Netherlands between 2000 and 2010, by age and type of admission

Age, years	Day clinic, n	Inpatient, n	Day, % deaths	Inpatient, % deaths	RR (95% CI) inpatient vs day clinic
1-year mortality					
60–64	541	646	3.3	24.6	7.40 (4.61 to 11.9)
65–69	1013	1301	6.2	28.1	4.51 (3.50 to 5.81)
70–74	2438	3505	8.1	33.1	4.10 (3.56 to 4.72)
75–79	4438	7673	9.9	36.9	3.72 (3.39 to 4.08)
80–84	5220	11 607	13.7	42.7	3.11 (2.89 to 3.33)
85–89	3756	10 408	17.5	47.4	2.72 (2.53 to 2.92)
90–94	1138	4456	26.4	55.2	2.09 (1.89 to 2.31)
95–99	157	904	33.8	60.3	1.79 (1.43 to 2.24)
Total	18 701	40 500	13.1	43.0	3.29 (3.16 to 3.42)
5-year mortality					
60–64	541	646	12.8	45.0	3.53 (2.79 to 4.47)
65–69	1013	1301	22.1	54.8	2.48 (2.19 to 2.81)
70–74	2438	3505	29.6	62.2	2.10 (1.96 to 2.25)
75–79	4438	7673	34.5	66.9	1.94 (1.86 to 2.03)
80–84	5220	11 607	41.6	71.4	1.72 (1.66 to 1.78)
85–89	3756	10 408	50.1	75.3	1.50 (1.45 to 1.55)
90–94	1138	4456	63.2	81.1	1.28 (1.23 to 1.35)
95–99	157	904	67.5	82.3	1.22 (1.09 to 1.36)
Total	18 701	40 500	39.7	71.1	1.79 (1.76 to 1.83)

RR, relative risk.

### Five-year mortality risk among patients with dementia

After 5 years, 65.4% of men and 58.5% of women had died following a first hospitalisation or day clinic visit for dementia. Five-year mortality risks for women and men showed similar results as 1-year mortality risks stratified by age and gender (table 2). Men had higher mortality risks compared with women (RR 1.12, 95% CI 1.10 to 1.13). Median survival time for women who died within 5 years was 331 days, IQR 70–851 and for men 246 days, IQR 51–697.

Similar findings were found across dementia subtypes. In total 14 548 of 23 566 women with AD (61.7%) died within 5 years. In men, this percentage was significantly higher; 9132 of 13 389 (68.2%) died (RR for men vs women: 1.10, 95% CI 1.09 to 1.12). In VaD, 62.5% of women and 70.6% of men died (RR for men vs women: 1.13, 95% CI 1.09 to 1.17). The overall age-adjusted RR for VaD versus AD was 1.06, 95% CI 1.05 to 1.08 (data not shown).

Five-year mortality risks were higher in patients following a first hospitalisation for dementia. Absolute mortality risks in patients admitted to the hospital were 71.1% (median survival time was 214 days, IQR 45–679) and 39.7% in patients visiting a day clinic (median survival time was 624 days, IQR 265–1069). The highest RR was found in patients aged 60–64 years; RR for inpatients versus patients visiting a day clinic: 3.53, 95% CI 2.79 to 4.47 (table 3).

### Comparison with mortality risks of the general population

**Figure 1:**
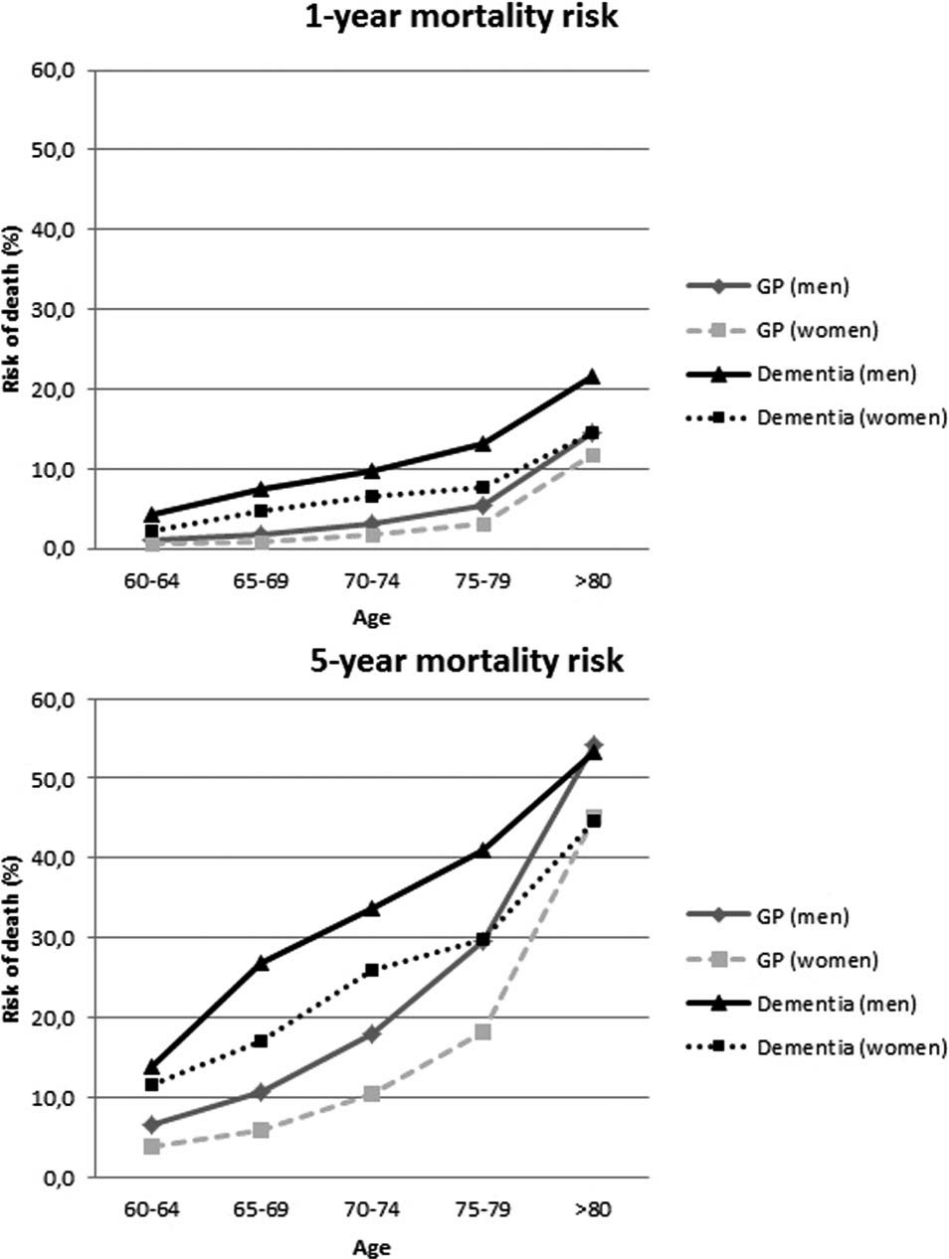
One-year and 5-year mortality risk in patients with a first day/memory clinic visit for dementia compared with the general population (GP, general practitioner).

Overall 1-year mortality risk in the general population until the age of 95 years was 7.5% for women and 8.9% for men. Absolute risks and RRs stratified by age and sex are presented in figure 1 and table 4. For women with a first day clinic visit for dementia, short-term mortality risk was 2.99 times higher (95% CI 2.84 to 3.14). For men, this risk was 3.94 times higher (95% CI 3.74 to 4.16).

**Table 4:** RRs for mortality in patients with a first day/memory clinic visit for dementia versus the general population stratified by age and sex

	Women		Men	
Age, years	Dementia vs general population		Dementia vs general population	
	RR	95% CI	RR	95% CI
1-year mortality				
60–64	3.56	1.61 to 7.87	4.01	2.30 to 6.97
65–69	4.84	3.24 to 7.22	4.24	3.14 to 5.72
70–74	3.90	3.18 to 4.79	3.17	2.66 to 3.79
75–79	2.48	2.16 to 2.84	2.42	2.15 to 2.73
>80	1.22	1.15 to 1.29	1.48	1.39 to 1.58
Total	2.99	2.84 to 3.14	3.94	3.74 to 4.16
5-year mortality				
60–64	3.06	2.18 to 4.28	2.15	1.60 to 2.87
65–69	2.85	2.34 to 3.48	2.51	2.18 to 2.89
70–74	2.49	2.27 to 2.74	1.87	1.72 to 2.03
75–79	1.64	1.54 to 1.74	1.38	1.31 to 1.46
80–84	0.99	0.96 to 1.02	0.98	0.95 to 1.01
Total	2.20	2.15 to 2.25	2.26	2.20 to 2.32

RR, relative risk.

Overall 5-year RR was 2.20 (95% CI 2.15 to 2.25) in first day clinic visit women as compared with the general population. For men, this risk was 2.26 (95% CI 2.20 to 2.32). According to 5-year age categories, mortality risks in patients with dementia compared with the general population decreased with increasing age.

### Comparison with mortality risks of other CVDs

**Figure 2:**
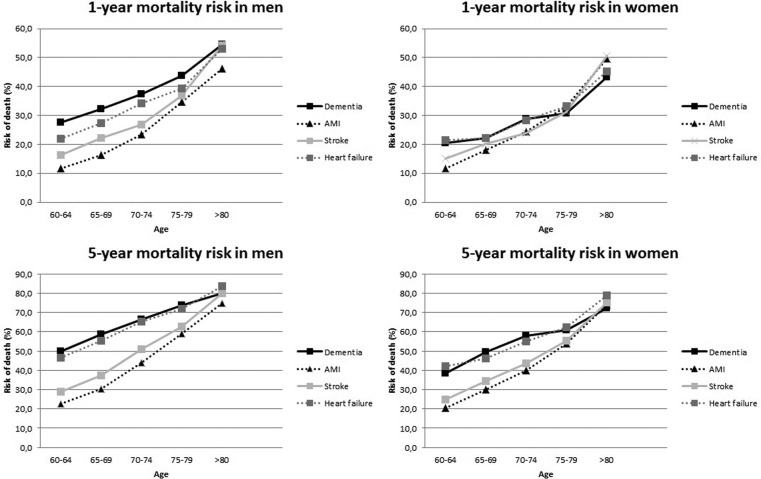
One-year and 5-year mortality risk in patients with a first hospitalisation for dementia compared with patients first hospitalisation for acute myocardial infarction (AMI), heart failure or stroke.

Absolute risks and RRs (patients hospitalised for dementia vs patients hospitalised for AMI, stroke and heart failure) stratified by age and sex are presented in figure 2 and table 5 with corresponding 95% CI. Patients with a first hospitalisation for dementia tend to have poorer 1-year mortality risks compared with patients hospitalised for CVDs. Risks were particularly higher in the youngest age groups (60–74 years) than the mortality risks following a diagnosis of AMI, heart failure and stroke. The highest RR was found in men with dementia aged 60–64 years compared with men with AMI (RR 2.37, 95% CI 1.93 to 2.91).

**Table 5:** RRs for mortality in patients with a first hospitalisation for dementia versus mortality risks after a first hospitalisation for AMI, heart failure or stroke, stratified by age and sex

Women	Age	Dementia vs AMI		Dementia vs heart failure		Dementia vs stroke	
	Years	RR	95% CI	RR	95% CI	RR	95% CI
1- year mortality	60–64	1.76	1.29 to 2.41	0.96	0.72 to 1.26	1.35	1.00 to 1.83
	65–69	1.24	1.00 to 1.52	1.00	0.83 to 1.22	1.10	0.91 to 1.34
	70–74	1.18	1.04 to 1.33	1.01	0.91 to 1.12	1.20	1.07 to 1.34
	75–79	0.93	0.84 to 1.01	0.93	0.87 to 1.00	0.98	0.91 to 1.06
	>80	0.87	0.83 to 0.91	0.96	0.93 to 0.99	0.85	0.83 to 0.88
	Total	1.24	1.19 to 1.29	1.05	1.02 to 1.08	1.07	1.04 to 1.10
5-year mortality	60–64	1.88	1.52 to 2.32	0.92	0.77 to 1.10	1.56	1.28 to 1.92
	65–69	1.65	1.45 to 1.88	1.07	0.96 to 1.19	1.44	1.28 to 1.62
	70–74	1.45	1.34 to 1.57	1.06	1.00 to 1.12	1.33	1.25 to 1.43
	75–79	1.13	1.07 to 1.20	0.98	0.94 to 1.01	1.10	1.05 to 1.15
	>80	0.97	0.95 to 1.01	0.91	0.90 to 0.93	0.96	0.94 to 0.97
	Total	1.37	1.33 to 1.40	1.01	0.99 to 1.02	1.16	1.14 to 1.19

AMI, acute myocardial infarction; RR, relative risk.

With respect to 5-year mortality prognosis, mortality risks in men with dementia admitted to the hospital were higher than the mortality risks following AMI, heart failure and stroke, particularly in the youngest age groups (highest RR was found in patients aged 60–64 years; RR for dementia vs AMI 2.21, 95% CI 1.94 to 2.52, and RR for dementia vs stroke 1.72, 95% CI 1.50 to 1.98, respectively). In women, risks were comparable to patients with heart failure, and were higher compared with AMI and stroke (highest RR was found in patients aged 60–64 years; RR for dementia vs AMI 1.88, 95% CI 1.52 to 2.32). Overall, RRs decreased with increasing age.

## Discussion

The present study, using a nationwide cohort of 59 201 patients with dementia, provides age-specific and sex-specific estimates on 1-year and 5-year risk of mortality. Men had an increased 1-year and 5-year risk of dying compared with women. Short-term mortality risks in patients visiting a day clinic were three to four times higher as compared with the general population. The risks among admitted patients even exceeded those observed in patients hospitalised with CVDs. AD and VaD had comparable mortality risks.

Several other studies also showed high overall mortality risks in dementia patients, ranging from 51.1% to 82% according to long-term prognosis.^6^ ^26–28^ These studies did not compare the results with other disease-specific subpopulations, thus making it hard to interpret the severity of the mortality risks. Some studies did make comparison with the general population and found comparable RRs (ranging from 2.0 to 3.7).^26–28^

Studies that analysed differences in mortality risks according to sex showed inconsistent results, although there is a general tendency towards higher mortality risks in male patients.^4^ ^10^ ^15^

Several other studies showed increased mortality risks with increasing age among patients with dementia, but a decreased RR with increasing age (ranging from a 3-fold to 6-fold higher risk in those aged <80 years to 1.5-fold to 3-fold higher risk in those aged ≥85 years) when compared with the general population.^27^ ^28^ An explanation could be that dementia at a younger age is of a more severe and progressive type than at an older age, leading to increased mortality.^27^ ^28^

Literature with respect to dementia subtypes is still inconclusive. Some studies have found higher risks with VaD compared with AD.^15^ ^27^ ^29^ We found comparable mortality risks among patients diagnosed with either AD or VaD in accordance with other studies that also demonstrated no differences in mortality.^13^ ^14^ ^30^ ^31^ It might be argued that this is due to a lack of power of studies showing no differences, but this is not an issue for the current study given the large size of the study population. Furthermore, inconsistency across the studies cannot be explained by differences in patient selection.

### Strengths and limitations

The strengths of the study are the large sample size, the complete follow-up of all included patients and the comparison with several other diseases to put mortality prognosis into perspective. A review of the literature confirms that the present study is one of the largest cohorts of patients with dementia. The validity of the linkage of different registries in the Netherlands has been demonstrated to be high.^32^ ^33^ Another strength is the high validity of the ICD-9 code to identify patients with dementia in the Dutch HDR, which we showed in a previously performed study.^21^ Furthermore, we had the opportunity to use data from other cohorts to compare mortality risks of patients with dementia to other diseases affecting the elderly so as to put these risks into perspective. Positive predictive values for the use of ICD-9 codes to identify these patients have shown to be acceptable.^34^

A limitation of this study is that except for absolute mortality risks according to age, sex, setting and type of dementia, other patient characteristics were not taken into account (eg, level of cognitive decline and severity of dementia and comorbidity). Therefore, we cannot conclude that the differences in age-specific and sex-specific prognosis between dementia and CVD can be entirely attributed to the dementia condition. Such a causality reasoning was beyond the scope of this particular study. We meant to report age-specific and sex-specific mortality rates of patients with dementia and its two most common subtypes. Although comparison with other external cohorts is possible given several available characteristics of the study population (eg, distribution of age, sex, diagnoses and hospital setting), this comparison will be limited by the lack of information on the aforementioned other factors.

Furthermore, generalisability of results is restricted to patients with dementia visiting a hospital. This means that results are applicable to approximately 22–30% of the patients with dementia in the Netherlands based on referral rate and incidence of the disease.^35^ ^36^

### Clinical implications

Given the poor prognosis of dementia (independent of type), especially found in men as well as in younger patients as compared with patients hospitalised for CVDs, we urge for more awareness of timely and proper management of patients with dementia in daily practice. The results may facilitate answering difficult questions concerning decision-making and advance care planning by patients, clinicians and carers. Furthermore, it stresses the urgent need for further research that will ultimately result in improvement of the poor prognosis in patients with dementia. This includes aetiological studies as well as studies focusing on prevention and treatment.

## Conclusion

In conclusion, this nationwide study showed that dementia has a poor prognosis, even poorer than commonly thought. One-year mortality risks were three to four times higher in patients visiting a day clinic compared with the general population. Mortality risks of patients with dementia admitted to the hospital even exceeded those following CVDs. The results of this study may facilitate answering difficult questions concerning decision-making and advance care planning in daily practice.
